# Preservation of the infected thoracic aortic endograft with thoracoscopic drainage and continuous irrigation

**DOI:** 10.1007/s11748-018-0893-2

**Published:** 2018-01-30

**Authors:** Fumiya Yoneyama, Fujio Sato, Hiroaki Sakamoto, Yuji Hiramatsu

**Affiliations:** 1 Department of Cardiovascular Surgery, University of Tsukuba, 1-3-1 Amakubo, Tsukuba, Ibaraki 305-8558 Japan; 2 Department of Cardiovascular Surgery, Tsukuba Medical Center, Tsukuba, Ibaraki Japan

**Keywords:** Endograft infection, Thoracoscopic operation, Endograft preservation

## Abstract

The gold standard for aortic endograft infection includes the excision of infected endograft, debridement, and reconstruction. However, these methods are not always the best option for patients with poor clinical status. We assessed the suitability of alternative methods for managing aortic endograft infection. The patient was a 72-year-old man whose previous abdominal surgeries provoked recurrent cholangitis. The patient had also undergone thoracic endovascular aortic repair (TEVAR). One month after the TEVAR, he was readmitted with high-grade fever and diagnosed with endograft infection. Due to his frail condition, we chose a less invasive and conservative strategy; thoracoscopic drainage with endograft preservation, followed by continuous irrigation. He recovered well, and has survived more than 2 years after the drainage procedure. In unstable patients or those with severe comorbidities who cannot tolerate endograft excision, thoracoscopic drainage with endograft preservation is less invasive, and can be a bridging or temporary solution.

## Introduction

Endograft infection is one of the most serious complications after thoracic endovascular aortic repair (TEVAR) with reported incidence of 0.2–5.0% [1]. Complete excision of the infected endograft, aggressive debridement, and aortic reconstruction is an optimal but invasive countermeasure to infection; it is, therefore, not applicable in all cases, and endograft preservation with antibiotics can be considered as a bridging therapy until excision of the infected endograft is performed in a more stable clinical condition [2]. Regarding the approach for drainage, thoracoscopic maneuver is less invasive than aortic surgery with thoracotomy in terms of giving less pain and greater patient acceptance postoperatively [3]. We present a case with endograft infection after TEVAR, who was treated with thoracoscopic drainage followed by continuous irrigation.

## Case report

**Fig. 1:**
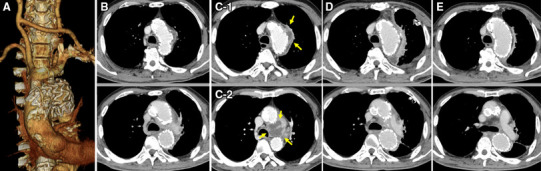
Contrasted computed tomography. **a** Three-dimensional image of a 2-debranch TEVAR. **b** Endograft infection was not demonstrated on admission. **c-1** Contrast enhancement around the endograft and fluid collection inside the remnant aneurysm. **c-2** Fluid filled cavity (roughly the size of a chicken egg) between aortic arch and pulmonary artery. **d** Postoperative 1 month. **e** Postoperative 2 years. No recurrence of endograft infection after thoracoscopic drainage and irrigation

**Fig. 2:**
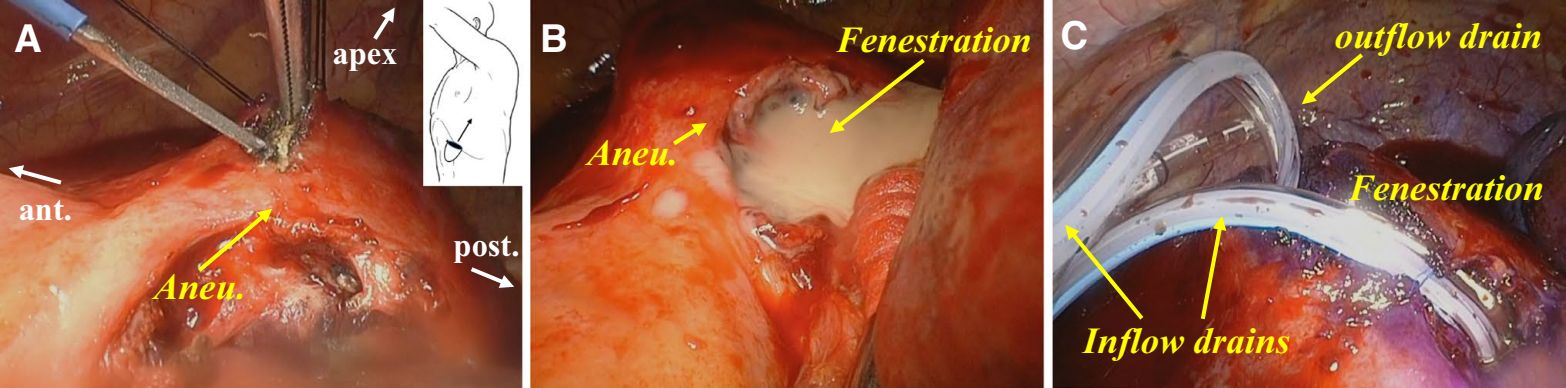
Thoracoscopic drainage surgery. **a** Incision on the lateral side of aneurysm with scalpel. **b** Purulent fluid erupting from fenestration. **c** Setting drainage tubes after drainage. *Aneu* aneurysm, *ant* anterior, *post* posterior

The patient was a 72-year-old man, whose previous surgical history included: intestinal repair for its rupture resulting from a traffic accident at 40 years of age, cholecystectomy for choledocholithiasis at 62 years, and pancreatoduodenectomy for pancreatic neoplasms when he was 63 years old. Although these previous abdominal surgeries had provoked recurrent cholangitis, his cholangitis had been treated well and under control in those days. We subsequently considered the intervention for thoracic aortic aneurysm, and presented him with the surgical choices: aortic surgery or thoracic endovascular aortic repair (TEVAR). He chose TEVAR because of its less invasiveness, and then we proceeded with TEVAR, with right common carotid–left common carotid–left subclavian artery bypass (Fig. 1a). He recovered well and was discharged without any complication. One month after the TEVAR, he was readmitted with high-grade fever and diagnosed with a recurrence of cholangitis. *Escherichia coli* was detected in his blood culture. Computed tomography (CT) on admission did not demonstrate endograft infection (Fig. 1b). Although the patient was started on cefozopran (3.0 g/day), he remained febrile (38.0–38.5 °C); his white blood cell (WBC) count was 15,000–20,000/µL and C-reactive protein (CRP) level was 15–20 mg/dL for more than 7 days. Repeated CT revealed contrast enhancement around the endograft, fluid collection inside the remnant aneurysm (Fig. 1c-1) and a fluid cavity (roughly the size of a chicken egg) between the aortic arch and pulmonary artery (Fig. 1c-2). No endoleak was confirmed. We assumed that the pathogen of cholangitis spread via blood stream, which resulted in endograft infection. Endograft infection might induce endoleak, endograft migration or aortic rupture. Although his cholangitis might be on active phase, we had priority to treat endograft infection initially.

Though the endograft excision with aortic reconstruction was optimal, we considered that the patient could not tolerate invasive aortic surgery with thoracotomy. His activity of daily life (ADL) was maintained at low level; he could communicate with others properly and did not have cognitive dysfunction such as dementia. However, he needed help for eating, changing clothes, and for the use of toilet, in daily life. He could walk a little but basically was on wheel chair during daytime. He suffered from severe chronic obstructive pulmonary disease, and treated with β2 stimulator inhalation. Pulmonary functional test revealed 45% of forced expiratory volume 1 s % (FEV 1.0%) and 0.9 L of forced expiratory volume 1 s (FEV 1.0). He also had septic cholangitis, with relatively high Euroscore II of 15.6. We also considered replaced-graft reinfection due to active and smoldering cholangitis. In addition, because of his abdominal surgical history, omental coverage was not an option. Therefore, we chose a less invasive and conservative strategy; thoracoscopic drainage with endograft preservation, followed by continuous irrigation.

Using a thoracoscopic surgical system through several access ports, we made an incision on the lateral side of the distal arch aneurysm (Fig. 2a) and created a 5-cm-length fenestration, which extended to the sub-aortic bulging area (Fig. 2b). The purulent fluid, which *E. coli* was cultured on later, erupted from the fenestration. We performed irrigation with copious saline containing gentamycin, in addition to crystal violet for gram-positive bacteria in case of polymicrobial infection. Two outflow drains (28Fr Argyle™ thoracic catheter and 24Fr Trocar catheter, Covidien, Dublin, Ireland) were placed on the diaphragm and at the apex of the left lung. Two inflow drains (10Fr BLAKE^®^ Silicone Drains, Ethicon, Newark, USA) (10Fr Nelaton catheter, Terumo, Tokyo, Japan) were placed lying on the fenestration (Fig. 2c).

The irrigation–drainage system was constructed postoperatively. Gentamycin, which is effective in gram-negative rod infection, was empirically diluted in 1000–2000 mL normal saline/day, and did not exceed the intravenous maximum dose (240 mg/day). By postoperative day (POD) 30, the abscesses had diminished upon CT imaging (Fig. 1d). His WBC count was normalized and CRP level stayed less than 0.03 mg/dL. *E. coli* was not cultured in the drainage fluid and blood, therefore we removed the inflow and outflow chest tubes on POD 32. After confirmation of no recurrent infection following drain removal, we decided to alter an intravenous cefozopran to oral amoxicillin (1.0 g/day) on POD 34. He recovered well and discharged on POD 92 via a rehabilitation hospital. In case of the next step as radical treatment such as total aortic arch replacement, we closely monitored his cholangitis and enhanced his ADL with rehabilitation. Oral amoxicillin was finished on POD 180 with a normal range of WBC count and CRP level. At current follow-up, he is now 28 months after drainage with no sign of recurrent infection including endograft infection and cholangitis (Fig. 1e).

## Discussion

Endograft infection after TEVAR is a critical complication with high morbidity and mortality rates [1]. Contrasted CT, gallium scintigraphy and positron emission tomography would be useful to identify the endograft infection. Although complete excision of the infected endograft is optimal, open radical surgery is associated with a high perioperative mortality (4–11%) and morbidity (35–67%) [1, 2, 4]. When conventional treatment requiring endograft excision is precluded, conservative treatment such as endograft preservation with antibiotic administration might be considered.

There has been much discussion on the management of endograft infection, and it is agreed that antibiotic administration must be given as a basic, first line of treatment. Fatima et al. reported that the most commonly identified organisms in the aortic wall were *Staphylococcus aureus* (63%) including methicillin-resistant *Staphylococcus aureus*, followed by *Streptococcus* and *Pseudomonas* in 24 infected endograft patients [4]. Fourteen patients (58%) among them suffered from polymicrobial infections, therefore broad-spectrum antibiotics should be selected. They concluded that there was no consensus or data of the appropriate antibiotic dose, interval, and duration. In this respect, broad-spectrum, life-long, and maximum-dose antibiotics should be considered. Additionally, we must also control primary infection as well as endograft infection to prevent its relapse.

Additional procedures supported by antibiotics, such as drainage, debridement, and irrigation, have recently demonstrated enhanced outcomes [5–7]. Cernohorsky et al. reported favorable outcomes with 5 of 6 patients treated conservatively with debridement and irrigation. They suggested that there could be room for conservative treatment when patients could not tolerate invasive treatment such as explant surgery [5]. Moulakalis et al. reported three cases of endograft infection that were successfully treated with endograft preservation by surgical debridement or percutaneous drainage [6]. However, these reports did not disclose the long-term outcomes and could not be considered as a conclusive treatment. Moulakalis et al. later performed a meta-analysis with a total of 55 patients who had previously undergone endograft preservation as reported in 41 studies. The in-hospital mortality in endograft preservation was 42.0% up to 81.8% in the follow-up period, and significantly worse than endograft excision. They mentioned that endograft excision remained the gold standard for endograft infection, whereas endograft preservation remained a temporary or bridging solution.

However, it should be noted that the candidates for endograft preservation are mostly of an unstable condition with severe comorbidity. They may not be able to tolerate invasive treatment such as aortic surgery with thoracotomy. When we chose the endograft preservation with irrigation as conservative treatment, we have basically two approaches, including open thoracotomy or thoracoscopic surgery. Open thoracotomy might be optimal for its clear field of view and maneuverability in order to eliminate and decorticate the abscess completely. In addition, we can deal with unintentional intraoperative bleeding immediately. On the other hand, thoracoscopic surgery is another approach and generally considered as a less invasive maneuver. Chan et al. reported that thoracoscopic surgery allows equally effective irrigation and decortication for empyema as thoracotomy, which gives less pain and greater patient acceptance [3]. This will be important especially for the patient with unstable condition and severe comorbidity. On the basis of this concept, our strategy of appropriate perioperative antibiotics administration, thoracoscopic drainage for the preservation of the infected endograft and continuous irrigation will be more effective and favorable combination as “maximally” less invasive therapy.

Our technique is limited to “non-endoleak” cases especially. With absolute confirmation of no endoleak, debridement around the infected endograft and its subsequent preservation can be performed. In this respect, precise perioperative evaluation of endoleak is crucial, and we evaluated it here with contrasted CT. Guo et al. performed the meta-analysis of the efficacy of contrasted CT for endoleak detection, with 31 studies of 3853 EVAR patients. They mentioned that CT had a significantly higher proportion of endoleak detection than ultrasonography (US) and magnetic resonance imaging (MRI) [8]. Nevertheless, CT cannot detect the endoleak perfectly; therefore, the combination of contrasted CT, US, and MRI will be effective to detect endoleak more precisely. If the bleeding with endoleak occurs under the limited thoracoscopic view field, it will be difficult to control. In our case, the fluid cavity was close to the proximal landing-zone of the endograft. Endograft infection can induce an endoleak and migration. We should be attentive to the distribution of infectious site and the landing-zone. Although intraoperative bleeding is a disaster, conversion from thoracoscopic surgery to open thoracotomy might be acceptable as second-line choice. Therefore, precise perioperative evaluation with CT and other methods, cooperation of thoracic surgeon familiar with thoracoscopic maneuver, and cardiopulmonary bypass back-up during surgery are all essential.

## Conclusion

In endovascular infection, surgical conversion as a final treatment may be postponed, but it may not be possible to avoid in the end. In unstable patients or those with severe comorbidities who cannot tolerate endograft excision and aortic reconstruction, thoracoscopic drainage with endograft preservation followed by continuous irrigation can be a temporary or bridging solution in the growing endovascular era. A further study should be conducted to establish the efficacy of this strategy.
